# A Thermostable Dissolving Microneedle Vaccine with Recombinant Protein of Botulinum Neurotoxin Serotype A

**DOI:** 10.3390/toxins14120881

**Published:** 2022-12-16

**Authors:** Baohua Zhao, Zhiying Jin, Yunzhou Yu, Yue Li, Jing Wang, Wei Wan, Chenyi Hu, Xiaoyang Li, Yanwei Li, Wenwen Xin, Lin Kang, Hao Yang, Jinglin Wang, Shan Gao

**Affiliations:** 1 Hebei Key Laboratory of Animal Physiology, Biochemistry and Molecular Biology, College of Life Sciences, Hebei Normal University, Shijiazhuang 050024, China; 2 State Key Laboratory of Pathogen and Biosecurity, Institute of Microbiology and Epidemiology, Academy of Military Medical Sciences (AMMS), Beijing 100071, China

**Keywords:** botulinum neurotoxin serotype A, botulism, dissolving microneedle patch, bacteriostatic, vaccine stability

## Abstract

Background: As a Class A bioterrorism agent, botulinum neurotoxin serotype A (BoNT/A) carries the risk of being used by terrorists to cause mass poisoning. The microneedle (MN) patch has a great potential for application as a novel vaccine delivery method. The aim of this study is to develop a thermally stable, dissolving microneedle patch for the delivery of a recombinant protein vaccine using a recombinant C-terminal heavy chain of BoNT/A (Hc of BoNT/A, AHc) to prevent botulism. Methods: Fish gelatin, a natural non-toxic and bacteriostatic material, was selected as the microneedle matrix for the preparation of the dissolving microneedle vaccine. Subsequently, the mechanical performance, bacteriostatic properties, vaccination effect, and stability of the microneedle patches were evaluated using instruments such as the displacement-force test station and optical coherence tomography (OCT) scanner. Results: Fish gelatin matrix at high concentrations has good bacteriostatic properties, and excellent mechanical performance and vaccination effect, meeting the necessities of a vaccine. In both in vivo and in vitro neutralization experiments, MN vaccines containing different antigen doses achieved the same protective efficacy as subcutaneous vaccinations, protecting mice against 10^6^ LD_50_ of BoNT/A injected intraperitoneally. Thermal stability analysis of the MN vaccines revealed that the fish gelatin matrix protected the AHc vaccine from protein denaturation even after 7 days of storage at 37 °C and enabled the vaccine patches to maintain good immunogenicity and protective efficacy even after 6 months of storage at room temperature. Conclusion: In this study, we successfully prepared a bacteriostatic MN patch using a fish gelatin matrix that not only has a good vaccination effect, but also obviates the need for a cold chain for the AHc vaccine, providing the possibility of rapid, painless, and large-scale vaccination.

## 1. Introduction

Botulinum toxin, also known as botulinum neurotoxin (BoNT), is a toxic protein produced by *Clostridium botulinum* that acts on the human and animal nervous system, causing neurological dysfunction. BoNT has seven main serotypes, labeled A through G. The toxins that cause human botulism are mostly types A, B, E, and F [1,2,3]. Among them, BoNT/A is the most toxic; 1 g of dry powder BoNT/A can kill 1 million people or 200 billion mice [4]. BoNT/A, as a class A bioterrorist agent, has the risk of being used by terrorists to cause mass poisoning. For example, between 1990 and 1995, Aum Shinrikyo carried out three botulinum toxin attacks in Japan [5]. Consumption of easily contaminated foods, such as fermented soybean products or air-dried meat, is a common route for botulism. In Xinjiang, China, at least two thousand cases of foodborne botulism have occurred since 1949, and the causative toxin was mainly BoNT/A [6]. Due to the lack of accurate and rapid clinical diagnosis of BoNT poisoning and the absence of specific antidotes, its case fatality rate can be as high as 20–40% [7]. In the face of bioterrorism or foodborne poisoning caused by BoNT, vaccination is one of the most effective means to prevent and control poisoning and reduce the case fatality rate [8]. The current vaccines used to prevent BoNT/A poisoning are toxoid vaccines, DNA vaccines, and recombinant protein vaccines [9]. Among them, recombinant protein vaccines have high purity, good safety, and do not cause side effects such as allergic reactions, but they are less thermally stable and require cold chain storage and transport. The traditional vaccination method of needle injection not only fails to solve the above problems, but also brings many inconveniences, such as causing pain and bleeding at the vaccination site, requiring specialized medical personnel for vaccination, and generating sharps waste.

Microneedle (MN) patches, arrays of microneedles from 100–1500 µm in length, are an emerging vaccine delivery method that provides a good solution to these problems. MN patches have the advantages of being thermally stable, causing minimal pain, and being self-administered [10]. Microneedle patches can be classified into five types according to the drug transport method: solid microneedles, hollow microneedles, coated microneedles, dissolving microneedles, and hydrogel-forming microneedles [11]. The first three types of microneedles have the risk of retaining solid waste in the skin due to fracture [12], and the safety of emerging materials used in the preparation of hydrogel-forming microneedles, such as poly (methyl vinyl ether-co-maleic acid) (PMVE/MA) and methacrylated hyaluronic acid (MeHA), is yet to be confirmed [13]. Dissolving microneedles dissolve completely after penetrating the skin without generating sharps waste, which can reduce the risk of cross-contamination [14]. Improving the stability of the vaccine and achieving long-term storage at room temperature [14,15,16] greatly reduces the cost of vaccine storage and transport.

The presence of abundant antigen-presenting cells in the skin makes the MN patch a highly promising vaccination modality [17]. However, MN patches may allow bacteria to enter the skin with the penetration of the microneedles when delivering vaccines, causing adverse reactions, such as infections and allergies [18]. Therefore, researchers have gradually begun trying to prepare antibacterial MN patches, for example, by adding antibiotics [19], graphene oxide [20], or silver nanoparticles [21] to achieve an antibacterial function of the patch. However, at the same time, problems of antibiotic abuse, lack of safety of the material, and high costs have arisen. To solve the above problems, we need to find new material for the preparation of antibacterial microneedle patches. Fish gelatin is obtained from the skin and bones of cold-water fish and has the advantages of widely available sources, weak antigenicity, good biocompatibility, safe and easy absorption, and a low price. It has been widely used in food, medicine, and cosmetics [22]. Fish gelatin has been reported to contain antimicrobial peptide components with antioxidant and antimicrobial activity [22]. Zhong, C. et al. prepared a gelatin-protocatechuic acid composite film with antioxidant and antibacterial activity, which effectively inhibited the growth of *E. coli* and *S. aureus* contaminated in beef [23]. Zhang, W. J. used chitosan-fish gelatin to prepare food packaging films for the antibacterial storage of food products [24]. Here, we try preparing antibacterial MN patches using fish gelatin.

BoNT/A is highly toxic, with a high lethality rate. To prevent BoNT/A botulism and solve problems in storage, transport, and vaccination of traditional vaccines, a novel thermostable microneedle vaccine of BoNT/A is in demand. In this study, the recombinant C-terminal heavy chain of BoNT/A (Hc of BoNT/A, AHc) plays the role of the receptor-binding domain. We mix AHc in a fish gelatin matrix to prepare microneedle patches with bacteriostatic properties and evaluate their various properties.

## 2. Results

### 2.1. Bacteriostatic Properties of the Microneedle Matrix

**Figure 1:**
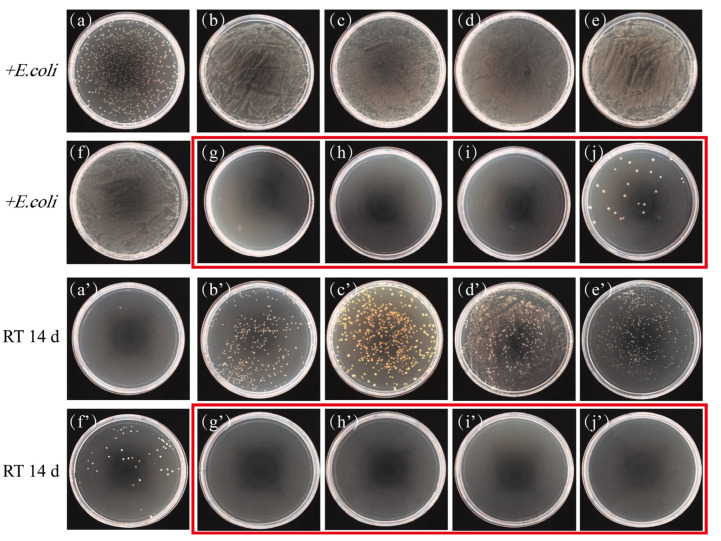
Bacteriostatic properties of the microneedle matrix. (**a**–**j**) Bacterial colonies formed in microneedle matrix solutions with *E. coli* added by agar dilution method. (**a’**–**j’**) Bacterial colonies formed in microneedle matrix solutions stored at room temperature (RT) for 14 d by agar dilution method. Samples with fish gelatin are outlined in red boxes. Microneedle matrix solutions: (**a**,**a’**) sterile water, (**b**,**b’**) 1% BSA, (**c**,**c’**) 10% sucrose, (**d**,**d’**) 35% nano hyaluronic acid + 1% BSA, (**e**,**e’**) 5% PVA + 1% BSA (10-fold dilution), (**f**,**f’**) 5% PVP-k17 + 1% BSA, (**g**,**g’**) 35% fish gelatin, (**h**,**h’**) 35% fish gelatin + 10% sucrose, (**i**,**i’**) 35% fish gelatin + 10% sucrose + 1% BSA, (**j**,**j’**) 10% fish gelatin + 10% sucrose + 1% BSA.

The bacteriostatic properties of the matrices were evaluated by observing the LB agar plates coated with the solutions of different matrices, with *E. coli* added or incubated at room temperature (22 ± 2 °C) for 14 d. When *E. coli* was added, three of the four matrix materials tested—nano hyaluronic acid, PVA, and PVP solutions—along with positive controls, BSA and sucrose (Figure 1b–f), produced more bacteria than the negative control (Figure 1a). In contrast, the number of bacteria in the fish gelatin matrix was significantly less than that of the negative control and even appeared sterile (Figure 1g–i). This indicates that fish gelatin has bacteriostatic properties that effectively inhibit the growth of *E. coli* even when the solution contains BSA and sucrose as nutrients. Comparing agar plates coated with 35% *w*/*v* and 10% *w*/*v* fish gelatin revealed that the higher concentration of fish gelatin could completely inhibit the growth of *E. coli* (Figure 1i,j) and was thus more effective at inhibiting bacteria. The same solutions without *E. coli* were plated and left at room temperature for 14 d. Colonies grew on plates coated with nano hyaluronic acid, PVA solution, and PVP solution (Figure 1d’–f’), with the number of bacteria in nano hyaluronic acid and PVA solution significantly higher than that in the positive control (1% BSA, Figure 1b’), indicating that these two substrate materials actually promote bacterial growth. The solution in the fish gelatin matrix did not grow bacteria regardless of the concentration of fish gelatin and whether BSA or sucrose were added (Figure 1g’–j’).

### 2.2. Microneedle Patches

#### 2.2.1. Preparation and Characterization of the Microneedle Patches

**Figure 2:**
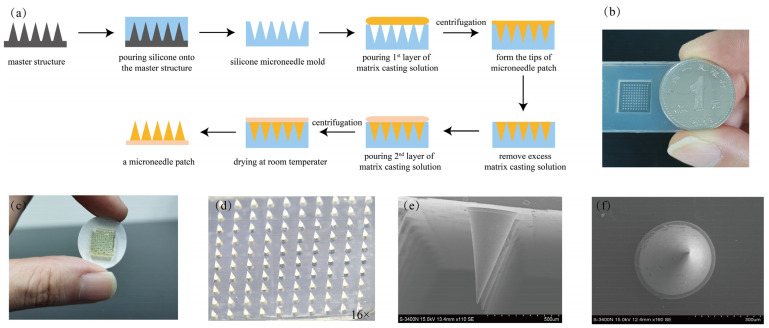
Preparation and characterization of the microneedle patch. (**a**) Diagram of the preparation process of the microneedle patch. (**b**) The silicone microneedle mold used to prepare the microneedle patches. (**c**) Overall view of the dissolving microneedle patch. (**d**) View of the microneedle array (10 × 10, 100 needles per patch) under stereoscopic microscope. Electron microscopy giving: (**e**) side view of microneedles (needle length of 650 μm) and (**f**) top view of microneedles (bottom surface diameter of 360 μm).

Dissolving microneedle patches with fish gelatin as the main matrix were successfully prepared by the micro-molding method using two centrifugations, as shown in Figure 2a. The results were observed with the naked eye, light microscopy, and a scanning electron microscope as shown in Figure 2b–f. The microneedle array was complete, with each patch containing 100 conical microneedles of 650 μm in length and 360 μm in bottom surface diameter. The distance between the microneedles was 360 μm.

#### 2.2.2. Mechanical Performance of the Microneedle Patches

**Figure 3:**
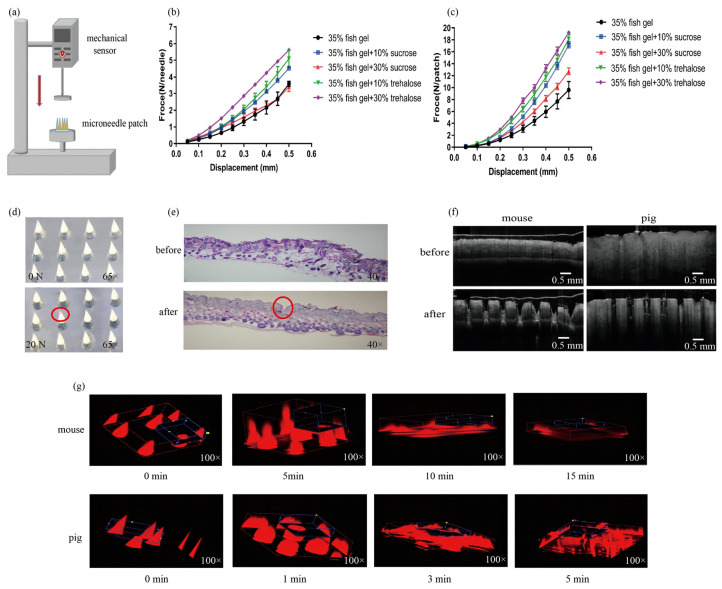
Mechanical performance of microneedle patches and their penetration and dissolving in the skin. (**a**) Diagram of the displacement-force test station. (**b**) Displacement-force for single microneedles (mean ± SEM, *n* = 5, one-way ANOVA, *p* > 0.05). Different color curves represent microneedle patches with different matrix materials. The horizontal axis is the distance the force gauge probe moves downward after contacting the microneedle, and the vertical axis is the pressure applied to each microneedle. (**c**) Displacement-force produced with microneedle patches (100 microneedles per patch, mean ± SEM, *n* = 5, one-way ANOVA, *p* > 0.05). (**d**) Microneedle patch before and after 20 N pressure applied. The red circle shows the microneedle that was only slightly deformed by the pressure of 20 N. (**e**) Pathological section of mouse skin before and after microneedle patch penetration. The red circle shows the area where the microneedle penetrated. (**f**) OCT scans of mouse skin and pig skin before and after microneedle patch penetration. (**g**) CLSM images of microneedle patches after penetration of mouse skin for 0–15 min and penetration of pig skin for 0–5 min. The red parts represent microneedles containing sulforhodamine B.

The mechanical performance of MN patches made from fish gelatin with different concentrations of sucrose or trehalose were measured using a displacement-force test station (Figure 3a). The mechanical performance of single microneedles and microneedle patches (100 microneedles per patch) were unaffected by the addition of sucrose and trehalose (*p* > 0.05; Figure 3b,c). Given the protective effect of sucrose on vaccine proteins and its low price, we used 10% *w*/*v* sucrose added to fish gelatin as an excipient for the preparation of subsequent microneedle vaccines. To investigate whether the microneedle patch of this formulation can penetrate the skin, we performed a pressure test and a skin penetration test. Under a pressure of 20 N, only the tip of the microneedle patch was slightly deformed (Figure 3d), indicating that the microneedle patch prepared from fish gelatin + 10% *w*/*v* sucrose has good mechanical performance and can withstand pressures above 20 N. The skin pathology section (Figure 3e) and OCT (Figure 3f) observations showed that the epidermal layer of mouse and pig skin were penetrated by microneedles, showing significant breaks. Thus, the microneedle patch has sufficient mechanical strength to penetrate mouse and pig skin and to achieve targeted delivery of the vaccine.

CLSM and OCT scanning were used to estimate the time required for the microneedles to completely dissolve after penetrating the skin. Microneedles gradually dissolved in the skin with time and were completely dissolved 15 min after penetrating mouse skin and 5 min after penetrating pig skin (Figure 3g; OCT graphs were shown as Figure S1 in Supplementary Materials). In the experiments that follow, we pressed microneedle patches with ≥20 N pressure for 15 min for the vaccination of mice.

### 2.3. Immunogenicity and Protective Efficacy of the Microneedle Vaccine

**Figure 4:**
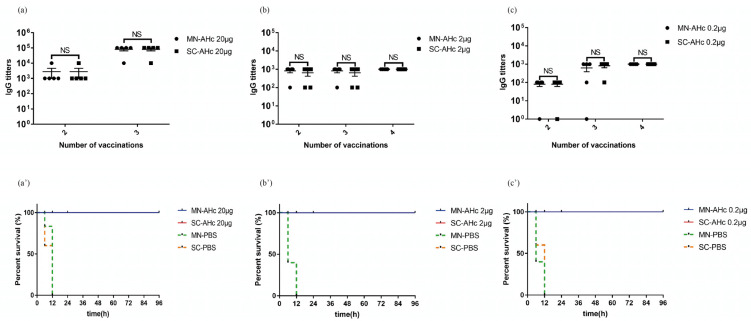
Immunogenicity and protective efficacy of microneedle vaccines. (**a**–**c**) Serum IgG antibody titers after AHc vaccination of mice (mean ± SEM, *n* = 5, *t*-test, *p* > 0.05, NS: no significant difference). The horizontal axis indicates the number of vaccinations. The vertical axis is the serum IgG antibody titers of mice. The doses of AHc vaccination were 20 μg, 2 μg, and 0.2 μg in (**a**–**c**), respectively. (**a’**–**c’**) Survival curves of mice after protective efficacy assay of 10^6^ LD_50_ BoNT/A. The horizontal axis is time. The vertical axis is the survival percentage of mice. Doses of AHc vaccination were 20 μg, 2 μg, and 0.2 μg in (**a’**–**c’**), respectively.

The vaccination effect of the MN patch versus the subcutaneous injection was compared by measuring the IgG antibody titer of mouse serum anti-AHc by ELISA (Figure 4a–c). Mice vaccinated with three doses of 20 μg AHc by both routes had serum antibody titers up to 10^5^, and mice vaccinated with four doses of 2 μg or 0.2 μg AHc had serum antibody titers up to 10^3^. In the protective efficacy assay, mice in both MN and SC groups were protected against up to 10^6^ LD_50_ BoNT/A, even at the lowest level of 0.2 μg of AHc, while all mice in the control group died, as shown in Figure 4a’–c’.

The presence of protective antibodies against BoNT/A in the mouse serum was detected using in vitro neutralization assays. The serum of mice vaccinated with 2 μg AHc (both MN and SC groups) fully protected mice against 10^2^ LD_50_ BoNT/A, even after 100× dilution. Both vaccination routes resulted in the production of neutralizing antibodies in mice. In summary, both microneedle patches and subcutaneous injections for the delivery of AHc produced the same immunological and protective effects. Therefore, we suggest that microneedle patches can replace subcutaneous injections for the delivery of AHc.

### 2.4. Stability of the Microneedle Vaccine

**Figure 5:**
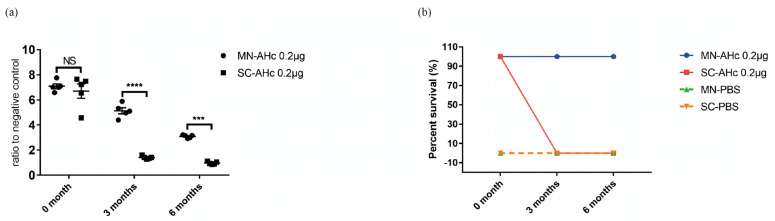
Stability of the microneedle vaccine. (**a**) Serum IgG antibody levels of mice after vaccination with AHc (0.2 μg per mouse) stored at room temperature. The horizontal axis is time of AHc storage. The vertical axis is the ratio of serum OD of mice after the last vaccination to the OD of the negative control (mean ± SEM, *n* = 5, *t*-test, NS: no significant difference, ****: *p* < 0.0001, ***: *p* < 0.0002). (**b**) Survival of mice after protective efficacy assay of 10^6^ LD_50_ BoNT/A. The horizontal axis is the time of AHc storage at room temperature. The vertical axis is the survival percentage of mice.

A protein thermal shift dye kit and a qPCR instrument were used to measure the Tm value of the AHc protein vaccine under different storage temperatures. When protein denatures and undergoes a conformational change, the signal of the fluorescent dye bound to the protein is continuously quenched. The changing curve of the AHc protein fluorescence signal can be measured by a qPCR instrument. The Tm value of AHc protein under different storage conditions was thus measured to ascertain whether storage conditions led to protein denaturation and to analyze the stability of AHc protein; this method is faster and easier than mouse experiments. The Tm value of the MN-AHc vaccine was 98.41 °C after 7 d at 37 °C, consistent with the positive control (liquid AHc vaccine stored at 4 °C for 7 d), indicating that the protein stability of the MN-AHc vaccine at 37 °C matched the protein stability at 4 °C for 7 d. In contrast, the Tm value of the liquid AHc vaccine decreased to 48.73 °C after 7 d at 37 °C, indicating the vaccine protein was denatured. Thus, the MN-AHc vaccine has good thermal stability. In addition, AHc vaccines stored at room temperature for an extended period of time were subjected to mouse experiments, and their long-term stability was evaluated with results of antibody titer and protective efficacy assays. Mice vaccinated four times with the MN-AHc vaccine stored at room temperature for 3 and 6 months produced antibody titers of 10^3^ and 10^2^, respectively (Figure 5a). Although antibody levels dropped to 10^2^ at 6 months, the mice were still fully protected against 10^6^ LD_50_ BoNT/A (Figure 5b). In contrast, none of the liquid AHc vaccines stored under the same conditions stimulated antibody production in mice or provided protective efficacy. The MN-AHc vaccine can thus be stored stably at room temperature for up to 6 months without any decrease in its protective efficacy, laying the foundation for the AHc vaccine to be free from cold chain storage and transport requirements.

## 3. Discussion

In this study, we successfully developed an MN-AHc vaccine patch with good thermal stability and bacteriostatic properties for the prevention of BoNT/A poisoning. This MN-AHc vaccine produces immunity consistent with subcutaneous injections and has two significant advantages over traditional vaccines. First, the microneedle vaccine does not require a skilled healthcare provider and can be administered by applying pressure to the skin for just a few minutes without pain and bleeding. Second, the MN-AHc vaccine can be stored at room temperature for long periods of time, reducing the cost of expensive vaccine stockpiles. In anticipation of a suspected BoNT/A exposure, the low-cost and self-administrable MN-AHc vaccine enables rapid vaccination of large populations, minimizing damage while not burdening the normal operation of medical facilities.

Mustafa Kamal et al. added carboxymethylcellulose in gelatin-based dissolving microneedle patches which significantly improved the mechanical properties of microneedles [25]. However, the fish gelatin we used can form microneedles with good mechanical properties without the addition of excipient. In addition, unlike other common gelatin (mammalian gelatin), fish gelatin has low gel strength and a low gelling and melting temperature [26]. As a result, fish gelatin solutions are prepared without heat and do not solidify into gel at room temperature, which has great advantages in the preparation. In our research, the addition of sucrose or trehalose in fish gelatin did not significantly affect the characterization and mechanical performance of the microneedle patches, so we chose to add 10% (*w*/*v*) sucrose based on the fact that it is a cheap and common pharmaceutical excipient.

This study is the first to suggest that fish gelatin can be used to prepare bacteriostatic microneedle patches. Using 35% fish gelatin inhibited the growth of *E. coli* at room temperature for up to 14 d without additional additives. Microneedles enable vaccination by forming microchannels on the skin surface, and thus also impair the barrier function of the skin, which can lead to infection from bacteria entering the body through the microchannels. The fish gelatin bacteriostatic matrix can effectively reduce the risk of infection and also provides an ideal means to guarantee the sterility of microneedle vaccines during production, storage, and use. In addition, the fish gelatin microneedle vaccine retains good immunogenicity and protective efficacy when stored at room temperature for up to 6 months. This lays the foundation for the AHc vaccine to be free from cold chain storage and transport, effectively solving the problem of the high cost of AHc vaccine due to the high cost of the cold chain storage and transport system.

The human epidermis is approximately 0.05–0.15 mm thick [27] and the dermis, which is rich in nerve endings and blood vessels, is between 0.6–3 mm thick [28]; the MN patch with a needle length of 650 μm prepared in this study is able to target vaccine delivery to the human epidermis and upper dermis while barely stimulating the nociceptive nerves and blood vessels deep in the dermis, thus causing minimal to no pain and no bleeding. The dissolving microneedle vaccine we prepared could penetrate the skin of mice under a thumb pressure of 20 N and completely dissolved within 15 min of penetration. For pig skin, this time was reduced to 5 min. The moisture content of pig skin has been reported to be about 68.26–80.31% [29], while that of mouse skin is about 60% [30]. The results presumably reflect that the moisture content in skin is closely related to the dissolving rate of microneedles. Although human skin has a similar structure to pig skin [31], moisture content differs considerably between them. Microneedle vaccines are usually administered to the human forearm, where the skin moisture content is about 30–40% [32]. Therefore, we predict that it will take more than 15 min for this microneedle vaccine to dissolve in human skin. In addition, during the experiments, we unexpectedly found that the temperature of the finger applying pressure can affect the dissolving speed of the microneedle. Future work to develop a microneedle vaccine delivery system that is insensitive to external influences, such as finger temperature, and to standardize the administration of microneedle vaccines by standardizing the applied pressure and time will be worthwhile.

## 4. Conclusions

This study is the first to successfully develop a dissolving microneedle patch with bacteriostatic properties using fish gelatin and sucrose as the main matrix, and to apply it to the delivery of the AHc vaccine. We show that fish gelatin in high concentration has good bacteriostatic properties that allow the microneedle matrix to be stored sterile at room temperature for extended periods, solving the problem of microneedle vaccines being easily contaminated during preparation and storage, and effectively improving the safety of microneedle vaccines. We also show that the microneedle vaccine has good mechanical performance and can withstand a pressure of more than 20 N. It can effectively penetrate the skin and deliver the target dose of the AHc vaccine. Once the microneedle penetrates the skin, it takes only 5–15 min to dissolve, leaving no residual sharps waste. Subsequent animal experiments demonstrated that the immune response induced by dissolving MN-AHc vaccine in mice did not differ significantly from that of subcutaneous AHc injection. Furthermore, the MN-AHc vaccine was effective in mice against 10^6^ LD_50_ BoNT/A even after 6 months of storage at room temperature. These findings indicate that this dissolving MN-AHc vaccine has good bacteriostatic properties, immunogenicity, and thermal stability, providing a novel and powerful tool for the prevention of BoNT/A poisoning and bioterrorism attacks.

## 5. Materials and Methods

### 5.1. Bacteriostatic Properties of Different Microneedle Matrix Solutions

Three materials commonly used in the preparation of dissolving microneedle patches, nano hyaluronic acid (Bloomage Biotechnology Corporation Limited, Shandong, China), polyvinyl alcohol (PVA) (Sigma 360627, St. Louis, MO, USA), and polyvinylpyrrolidone (PVP, Tianjin Boai NKY International Ltd. 9003-39-8, Tianjin, China) were compared with fish gelatin for their bacteriostatic properties. Matrix solutions were supplemented with 1% *w*/*v* bovine serum albumin (BSA, Amresco 0332, Washington, WA, USA) to simulate the vaccine protein. Sterile water was used as a negative control, while 1% *w*/*v* BSA and 10% *w*/*v* sucrose were used as positive controls (more details in Table 1). For each solution above, 1 mL was added to 4 mL of sterile water, followed by 50 μL of *E. coli* at a final concentration of 10^6^ CFU/mL, and incubated for 6–8 h at 37 °C with shaking at 180 rpm. Subsequently, 100 μL of each solution was evenly coated onto LB agar plates, incubated at 37°C for 48 h, and then observed using a colony counter (Synbiosis Proto, Cambridge, UK). In addition, each of the above solutions without added *E. coli* were incubated at room temperature (22 ± 2 °C) for 14 d and coated on LB plates for culture observation to compare the inhibition time of different substrates.

### 5.2. Microneedle Patches

Mice were used to assess microneedle vaccine efficacy, while mouse and pig skin were used to assess the ability of microneedles to break the skin surface. Female SPF-grade BALB/c mice at 6–8 weeks-old were purchased from Sipeifu (Beijing, China). Mice were housed in the SPF Animal Experiment Center of the Academy of Military Medical Sciences and fed sterile commercial mouse food (Sipeifu, Beijing, China). All studies were approved by the Institutional Animal Care and Use Committee (IACUC) of the Academy of Military Medical Sciences, review number IACUC-DWZX-2020-046 (approved on: 7 May 2020). Pig skins were purchased from Sun mart (Beijing, China).

The AHc vaccine was prepared by Yunzhou Yu. In brief, the AHc DNA fragment was subcloned into a pTIG-Trx expression vector. After *E. coli*, which contains pTIG-Trx-AHc, expressed AHc and was disrupted by sonication, the recombinant AHc protein was purified by conventional chromatographic columns, which included HiTrapTM SP FF, HiTrapTM Q FF, and HiTrapTM octyl FF. More details can be found in the reference [33].

#### 5.2.1. Preparation of MN-AHc Patch

The dissolving microneedle AHc vaccine (MN-AHc) was prepared using the micro-molding method with two centrifugations [34]. The general method involved adding the AHc microneedle tip matrix to a silicone mold (provided by Shanghai Jiaotong University) and centrifuging at 3000 rpm for 10 min (Beckman Coulter, 21R Centrifuge, San Pablo, CA, USA) to allow the tip matrix to enter the micropores of the mold, after which the residual matrix from the surface of the mold was scraped off. The MN patch backing was prepared by centrifuging 100 μL of the matrix solution without AHc in the same manner. After drying at room temperature (25 ± 2 °C), microneedle patches were carefully peeled from the molds and mounted on 21 mm-diameter circular tape (Simaier, Harbin, China) to obtain dissolving microneedle vaccines containing 20 μg, 2 μg, 0.2 μg, or 0 μg AHc per patch. In addition, a control patch with no AHc added to the tip matrix was created.

In addition, MN patches with matrix formulations that varied in type and concentration of sugar (and without AHc) were prepared to investigate the effect of sucrose and trehalose on the mechanical performance of microneedle patches: 35% *w*/*v* fish gelatin (Sigma G7041, St. Louis, MO, USA); 35% *w*/*v* fish gelatin + 10% *w*/*v* sucrose (Sigma V900116, St. Louis, MO, USA); 35% *w*/*v* fish gelatin + 30% *w*/*v* sucrose; 35% *w*/*v* fish gelatin + 10% *w*/*v* trehalose (Sigma T9531, St. Louis, MO, USA); 35% *w*/*v* fish gelatin + 30% *w*/*v* trehalose. Finally, sulforhodamine B (Sigma 341738, St. Louis, MO, USA) at a final concentration of 1 mg/mL was added to a tip matrix of 35% *w*/*v* fish gelatin + 10% *w*/*v* sucrose to prepare microneedle patches for an evaluation of the needle dissolving rate.

#### 5.2.2. Mechanical Performance and Skin Penetration of Microneedle Patches

The force analysis of single microneedle and 100-needle MN patches under axial load was performed with a displacement-force test station (Mark-10, Copiague, NY, USA). The microneedle patch was placed on a rigid metal platform with needle tips facing upwards vertically, and the sensor probe approached the microneedle patch in the vertical direction at a speed of 0.1 mm/s. Displacement and force were recorded from the first contact of the sensor probe with the microneedle tip(s) and stopped when the sensor probe travelled 0.5 mm toward the patch backing. The displacement-force data were analyzed to compare the mechanical performance of microneedle patches prepared with different matrix formulations. Since the force required for different types of microneedle patches to penetrate the skin is less than 20 N [35,36,37], microneedle patches prepared from a matrix formulation with better mechanical performance were selected and an axial pressure of 20 N was applied to observe and evaluate the pressure they could withstand.

To confirm the penetration of microneedles into the skin, we observed skin through pathology sections and optical coherence tomography (OCT) scans (MDL, VivoSight DX, Kent, UK). Pathological sections were made from the skin of mice before and after microneedle penetration, and the epidermal layer of the skin was observed for breaks using an optical microscope (Olympus DP71, Tokyo, Japan). The OCT probe was placed on mouse skin and pig skin after microneedle penetration, and the skin was scanned and observed.

Microneedle patches with needle tips containing sulforhodamine B were pressed into mouse skin for 0, 5, 10, and 15 min, and into pig skin for 0, 1, 3, and 5 min. Subsequently, residual microneedles on the patch were observed with a confocal laser scanning microscope (CLSM, Olympus FV1000, Tokyo, Japan). Furthermore, the real-time in situ dissolving of microneedles in the skin at the above time points was observed by OCT scanner.

### 5.3. Evaluation of the Vaccination Effect of the Microneedle Vaccine

Mice were selected for vaccination and grouped into microneedle patch (MN) and subcutaneous injection (SC) groups, as shown in Table 2. 20 µg, 2 µg, 0.2 µg, or 0 µg AHc was delivered through MN or SC, respectively (*n* = 15). Mice in the MN group were dorsally shaved the day before vaccination. Each mouse was vaccinated three or four times, with 2 weeks between each vaccination. Blood was collected from the tail vein of mice 7 d after each vaccination. Blood samples were separated by centrifugation at 4 °C and 4000 rpm for 10 min to obtain the serum.

#### 5.3.1. Antibody Titer Assay

Serum anti-AHc titers were measured by ELISA for each group of mice [38]. AHc was first diluted to 3 μg/mL with an ELISA coating solution (diluted to 1×, Solarbio C1055, Beijing, China) and 100 μL per well was added to a 96-well ELISA plate (Thermo 442404, Waltham, MA, USA) and left overnight at 4 °C. On the following day, 350 μL PBST solution (PBS + 0.5% Tween 20) was added to each well with a plate washer (Tricontinent MultiWash III, San Pablo, CA, USA), and the plate was washed three times. Then, 350 μL of 3% BSA was added to each well and the plate was washed after incubation at 37 °C for 1 h. Mouse serum diluted in gradient (100 μL) was added as the primary antibody in each well at dilutions of 10^2^, 10^3^, 10^4^, and 10^5^ times, and incubated for 45 min at 37 °C. After washing the plate with PBST, 100 μL of 1:5000 diluted goat anti-mouse IgG-HRP (Solarbio SE131, Beijing, China) was added to each well as the secondary antibody, and the plate was washed after incubation at 37 °C for 30 min. In each well, 100 μL of color development solution (Solarbio PR1210, Beijing, China) was added; wells were kept in the dark for 10 min and then 100 μL of termination solution (Solarbio C1058, Beijing, China) was added. Optical density (OD) values at 450 nm were measured by a microplate reader (Thermo Multiskan MK3, Waltham, MA, USA). Serum samples from mice not vaccinated with AHc were used as negative controls. Samples from vaccinated mice (positive samples) meeting the condition (P_OD_−B_OD_) ≥ 2.1(N_OD_−B_OD_), where P_OD_ was the OD value of the sample, N_OD_ was the OD value of negative control sample, and B_OD_ was the OD value of the blank control, were considered positive. The antibody titer was expressed as the reciprocal of the maximum dilution of the sample giving a positive result.

#### 5.3.2. Mouse Serum Neutralization Assay

Sera from mice in groups 2, 4, 6, and 8 (Table 2) were collected 7 d after the last vaccination and used for the toxin neutralization assay; groups 4 and 8 were the control groups for MN and SC groups, respectively. The 100-fold diluted post-vaccination serum was mixed with equal volumes of BoNT/A at 10 LD_50_ and 100 LD_50_ and incubated at 37 °C for 30 min. Unvaccinated mice were injected intraperitoneally with 500 μL of the above serotoxin mixture (*n* = 5) and their survival was recorded.

#### 5.3.3. Protective Efficacy Assay

Two weeks after the last vaccination, 15 mice from each group were divided into three equal groups; each group was injected intraperitoneally with 500 μL of BoNT/A at 10^2^ LD_50_, 10^4^ LD_50_, and 10^6^ LD_50_, and their survival was recorded.

### 5.4. Stability Evaluation of the Microneedle Vaccine

Traditional liquid AHc vaccines and MN-AHc vaccines were placed at 37 °C for 7 d and protein Tm values were measured using a qPCR apparatus (Analytik Jena qTowerR3G, Germany) [39,40] to compare the stability of the two AHc vaccines. Liquid AHc vaccines stored at 4 °C for 7 d were used as a positive control, and all samples were set up in two replicate wells. The qPCR reaction system was as follows: 5 μL buffer, 12.5 μL sample, and 2.5 μL Dye (8×) (Thermo 4461146, Waltham, MA, USA). The qPCR program was as follows: Step 1 at 25 °C for 2 min; Step 2, melting curve was set from 25 °C to 99 °C and ΔT = 0.3 °C/s.

Mice were vaccinated on days 0, 14, 28, and 42 with a liquid AHc vaccine or MN-AHc vaccine (each patch contained 0.2 μg AHc, packaged in aluminized vacuum bags) stored at room temperature (25 ± 2 °C) for 3 and 6 months. Blood was obtained from the tail vein of mice 7 d after each vaccination, and serum was collected. Two weeks after the last vaccination, each group was injected intraperitoneally with 500 μL of BoNT/A at 10^6^ LD_50_ and their survival was recorded.

### 5.5. Statistical Analysis

Experimental data on the mechanical performance of the microneedles, the penetration of the microneedles, and vaccination-related assays were processed and plotted using GraphPad Prism 7 software. One-way analysis of variance (ANOVA) was used to assess whether the matrix formulation influenced the mechanical performance of the microneedle patches. For statistical analysis of antibody titers, *t*-tests were used. Only data with *p* < 0.05 were considered statistically significant in all experiments.

## Figures and Tables

**Table 1:** Formulation of microneedle matrix solutions.

No.	Matrix	Concentration (*w*/*v*)	Excipient	
			10% Sucrose	1% BSA
1	Fish gelatin	35%	−	−
2	Fish gelatin	35%	+	−
3	Fish gelatin	35%	+	+
4	Fish gelatin	10%	+	+
5	Nano hyaluronic acid	35%	−	+
6	PVA	5%	−	+
7	PVP	5%	−	+
8	Sterile water		−	−
9	Sterile water		+	−
10	Sterile water		−	+

PVA: polyvinyl alcohol; PVP: polyvinylpyrrolidone.

**Table 2:** Vaccination experiment groups.

No.	Vaccination Method	Dosage (µg)	Number of Mice per Group
1	MN	20	15
2	MN	2	15
3	MN	0.2	15
4	MN	0	15
5	SC	20	15
6	SC	2	15
7	SC	0.2	15
8	SC	0	15

MN: microneedle patch; SC: subcutaneous injection.

## Data Availability

The data presented in this study are available on request from the corresponding author.

## Supplementary Materials

The following supporting information can be downloaded at: https://www.mdpi.com/article/10.3390/toxins14120881/s1, Figure S1. Dissolution time of microneedles in skin (a) OCT scans of mouse skin after microneedles penetrated for 0–15 min. (b) OCT scans of pig skin after microneedles penetrated for 0–5 min.
