# Supplementary material for: A Thermostable Dissolving Microneedle Vaccine with Recombinant Protein of Botulinum Neurotoxin Serotype A
Source: Toxins (Basel). 2022 Dec 16;14(12):881. doi: 10.3390/toxins14120881 (PMC9781108; doi:10.3390/toxins14120881)
Supplement: Supplementary file 1 [file toxins-14-00881-s001.zip › toxins-2017241-supplementary.pdf]

toxins

MDPI

Supplementary Materials: A Thermostable Dissolving Microneedle Vaccine with Recombinant Protein of Botulinum Neurotoxin Serotype A

Baohua Zhao, Zhiying Jin, Yunzhou Yu, Yue Li, Jing Wang, Wei Wan, Chenyi Hu, Xiaoyang Li, Yanwei Li, Wenwen Xin, Lin Kang, Hao Yang, Jinglin Wang and Shan Gao

I. Figure for Supplemental Information

S1. Dissolution time of microneedles in skin.

Mouse skin was scanned after microneedles penetrated for 0, 5, 10 and 15 min by OCT to observe the real-time in situ dissolving of microneedles. Microneedles in pig skin were observed in the same way at the time of 0, 1, 3 and 5 min.The results show that microneedles were completely dissolved 15 min after penetrating mouse skin and 5 min after penetrating pig skin. It is the same as the result of confocal laser scanning microscope.

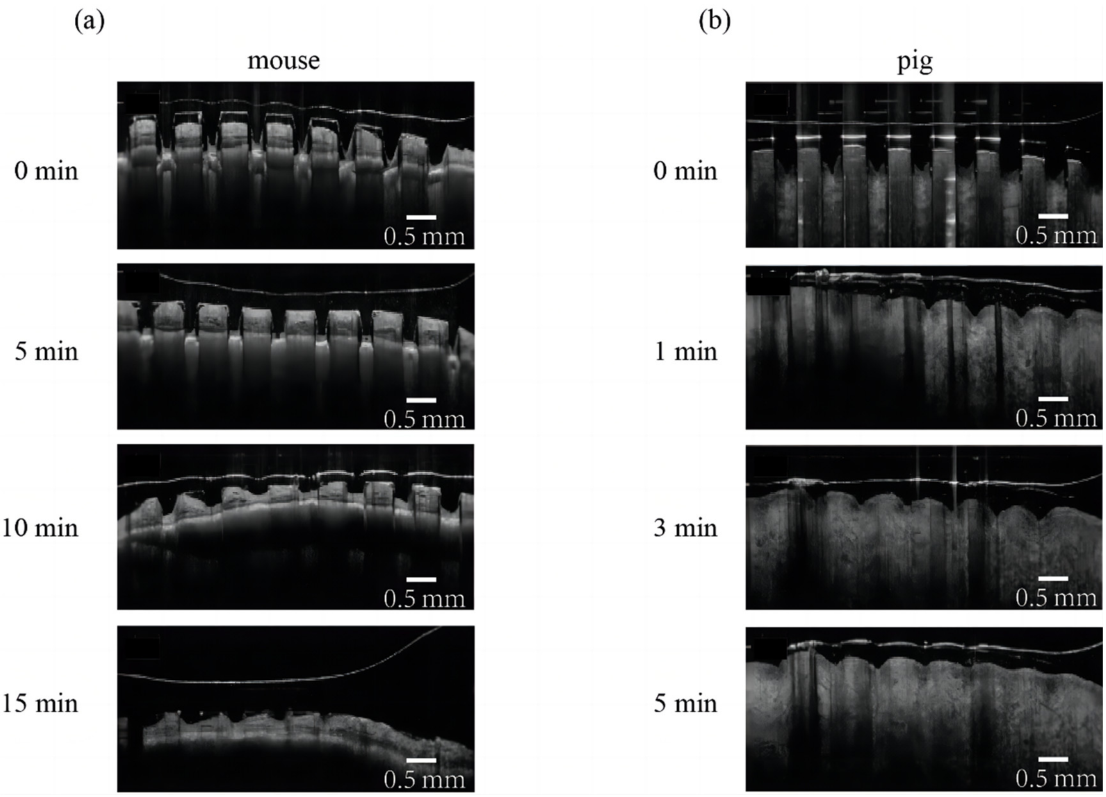

Figure S1. Dissolution time of microneedles in skin. (**a**) OCT scans of mouse skin after microneedles penetrated for 0-15 min. (**b**) OCT scans of pig skin after microneedles penetrated for 0-5 min
